# The Three-Dimensional Morphology and Distribution of CaS Inclusions in Continuous Casting Slab of Ni20Mn6 Steel

**DOI:** 10.3390/ma13173891

**Published:** 2020-09-03

**Authors:** Jing Chen, Jing Zhang, Shaobo Zheng, Jieyu Zhang

**Affiliations:** State Key Laboratory of Advanced Special Steel, Shanghai Key Laboratory of Advanced Ferrometallurgy, School of Materials Science and Engineering, Shanghai University, Shanghai 200072, China; Meanderzj@shu.edu.cn (J.Z.); zjy6162@staff.shu.edu.cn (J.Z.)

**Keywords:** calcium sulfide inclusions, solidification structure, macro and microsegregation, columnar-to-equiaxed transition

## Abstract

Calcium sulfide (CaS) inclusion with large and irregular shape is detrimental to the properties of steel. Understanding the shape and distribution of CaS inclusions in a continuous casting (CC) slab is of significance for improving the rolling properties. In this study, CaS inclusions were extracted from CC slab of Ni20Mn6 steel using the electrolytic extraction and investigated by scanning electron microscopy (SEM)-energy dispersive X-ray spectroscopy (EDX). The CaS inclusions morphologies vary with their locations in the CC slab and, thus, are classified into five categories. The thermodynamics calculated results showed that CaS inclusions precipitated at the end of solidification due to the microsegregation of sulfur and calcium in the interdendrite liquid and finally precipitated along the austenite grain boundary. The macrosegregation degree of solutes in different regions is one of the reasons that affect the size of CaS inclusion. The morphologies of CaS inclusion are affected by the solidification structure of slab and austenite grain boundary.

## 1. Introduction

Ni20Mn6 high-alloy steel, a precision alloy defined in national standard GB/T 37797-2019, is a functional alloy with controllable thermal expansion properties, low thermal conductivity, and high plasticity [1]. Its unique properties of high dimensional stability, high temperature resistance, and corrosion resistance mean it has an irreplaceable position in niche areas, such as ocean engineering, environmental engineering, astronaut, and aerospace engineering. Steel of good machinability and mechanical properties are required for producing thin walled parts, with very precise dimensions and complex shapes. Sulfide inclusions have been found a critical issue for Ni20Mn6 high-alloy steel, since the sulfide inclusions morphology, size, and distribution significantly affect the alloy properties [2,3,4,5].

Calcium sulfide (CaS) is a solid precipitate in liquid steel with high-melting point. Its solid particles are susceptible to collision and agglomerate into complex geometric shapes, which could cause the steel physical properties to deteriorate, especially when the particle is large and irregular [6,7]. The thermal expansion coefficient difference between CaS inclusions and steel matrix will cause stress concentration surrounding the inclusion during rolling process and causing the fatigue crack. The larger the size of inclusions, the larger the size of cracks, which accelerates the fatigue damage [8]. On the other hand, the CaS particles can be easily dissolved into acid, resulting in reducing the corrosion resistance of steel. Furthermore, calcium sulfides make steel prone to hydrogen-induced cracking. Therefore, it is necessary to understand the distribution and precipitation behavior of CaS inclusion in a casting slab.

Numerous studies have been devoted to understanding and controlling the morphology of sulfide inclusions. A classical work classified the morphology of sulfide inclusions into three types [9,10]: (1) Type I: randomly dispersed globular sulfides with a wide range of size, (2) Type II: sheet-like or rod-like fine sulfides, and (3) Type III: angular sulfides formed in deoxidized melts. Comparing the deformability of these three types sulfide inclusions, Type II sulfide inclusions can be deformed to a large extent during hot working and be arranged in a long string along the rolling direction, thereby reducing the impact toughness of steel and causing brittle fracture [11,12,13]. 

At present, studies on calcium sulfide inclusions mainly focused on the complex inclusions during the Ca-treatment. In Ca-treated steels, the calcium sulfide exists in different ways: (a) stable CaS in sulfur-rich steels [14]; (b) CaS shell as an outer layer of calcium aluminate [15,16]; (c) transient CaS formed immediately after calcium treatment and, then, transformed into CaO [17,18]; (d) MnS-rich sulfides containing some CaS [19,20]. Although so many works have been done in the past, the studies on the pure CaS inclusions are not enough, especially studies on the morphology of CaS inclusion has not been considered yet.

Studies on the connection of solidification structure and sulfide inclusions still focused on the size of dendrite structures instead of the macrostructure. Imagumbai et al. [21,22] studied the relationship between solidification conditions and the morphologies of sulfide inclusion in dendrite structure. However, the connection between sulfide inclusion and macrostructure of the whole transverse section of slab has not been intensively studied yet, especially in the columnar-to-equiaxed transition zone. 

This work firstly describes the morphology and distribution of CaS inclusions on the cross-sectional of the slab firstly. Then, thermodynamic calculation is carried out to analyze the formation mechanism of CaS inclusion. The combined effect of macrosegregation and solidification structure of slab on the CaS inclusion is discussed. 

## 2. Experimental Procedures

The production process comprises of 40t Electric Arc furnace, Argon Oxygen Decarburization (AOD), ladle furnace (LF) refining, followed by vacuum decarburizing (VD) refining, continuous casting, and rolling. The continuous casting describes the molten metal is solidified into semi-finished production for further rolling process [23]. In this study, a continuous casting slab sample of Ni20Mn6 steel was chosen from a vertical bending caster with 190 mm thickness. The chemical compositions of the continuous casting slab are listed in Table 1, where T.O is the total oxygen. 

**Figure 1:**
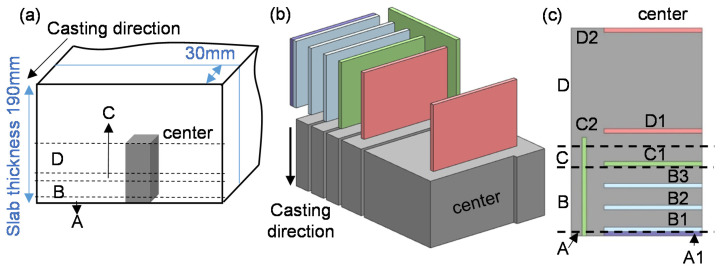
Schematic of sampling locations in casting slab. A is the chilled layer zone, B is columnar zone, C is columnar-to-equiaxed transition zone (CET zone), and D is equiaxed zone. (**a**) General view of the slab; (**b**) isometric view of slab; (**c**) top view of slab.

In order to distinguish different macrostructures of the slab, a 3 mm thickness of slab in the cross-section was etched in a saturated water solution of the picric acid at 75 °C for 2 min. The etched surface was observed using optical microscope. Using the carbon sulfur analyzer and inductively coupled plasma optical emission spectroscopy (ICP-OES, Thermo Scientific, Waltham, MA, USA) to analyze the content S and Ca in different regions, the degree of macrosegregation of S and Ca are obtained. A series of steel samples with 45 mm length, 30 mm width, and 2 mm thickness were cut along the thickness direction in order to analyze the three-dimensional morphology and the distribution of CaS inclusions. The sampling method is schematically shown in Figure 1 and Table 2.

To illustrate and distinguish the difference between each other, eight samples were named as A1, B1, B2, B3, C1, C2, D1, and D2.

**Figure 2:**
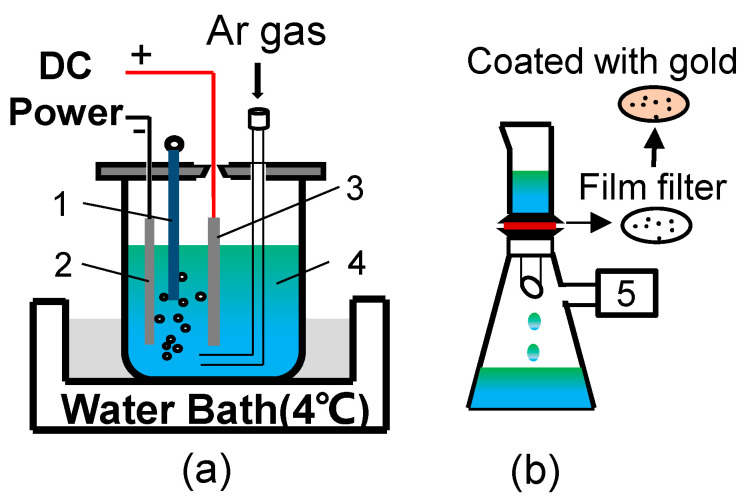
Schematic illustration of electrolytic process: (**a**) extraction; (**b**) filtration. 1. Thermometer, 2. Cathode, 3. Steel sample, 4. Electrolyte, 5. Vacuum pump.

The nonaqueous solution electrolysis method [24,25] was employed to accurately observe the three-dimensional morphology of sulfide. The schematic illustration of experimental device for electrolytic extraction is shown in Figure 2.

The steel samples were polished and later used as the anode, and a thin-platinum plate was used as the cathode. In this work, the dissolved sample weight during electrolysis was about 0.5 g, the electrolyte was composed of 56% Methanol, 40% Maleic anhydride, 4% tetramethylammonium chloride. During the electrolysis process, the power was supplied by a direct-current source and the voltage was set to 1.9 V, the electrolytic cell was placed in a water bath at 4 °C. To avoid oxidation of steel samples, the procedure of extraction was performed under the protection of argon atmosphere. After magnetic separation, washing, filtration with an open pore size of 3 μm, and drying, the remaining inclusions were separated from the steel [26]. Finally, the three-dimensional morphology and composition of the sulfide inclusions in the extracted residues were characterized by scanning electron microscope equipped with energy dispersive X-ray spectrometry (SEM-EDX, Tokyo, Japan).

## 3. Results

### 3.1. Macrostructure of Slab

**Figure 3:**
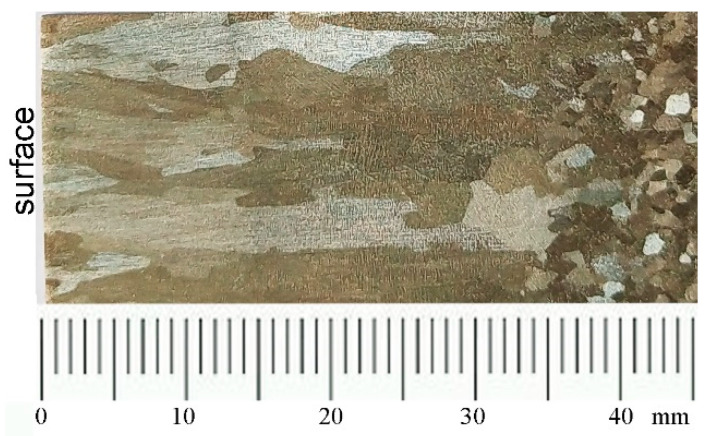
Macrostructure of continuously cast slab.

The macrostructure of slab includes chilled layer zone, columnar zone, columnar-to-equiaxed transition (CET) zone, and equiaxed zone. Figure 3 shows the macrostructure of casting slab transverse section revealed by macro-etching techniques. 

The width and area ratio of each zone was listed in Table 3.

A thin chilled zone forms at the slab surface immediately due to the intensive cooling nearby the mold wall. As the cooling process goes further, columnar crystals grow along the direction of the temperature gradient—preventing the growth of other crystals—and then develop to a columnar zone. When the supercooling in the front of solid–liquid interface of columnar zone continuous to increase, a large number of equiaxed crystals are formed, which hinders the further growth of the columnar crystals. The transition from columnar crystals to equiaxed crystals occurs. After that, the equiaxed crystals grow in the liquid ahead of the columnar zone. The growth of equiaxed crystals block the further advance of the columnar crystals’ growth and finally the CET zone takes place [27,28,29]. Then equiaxed zone develops in the slab center.

### 3.2. Three-Dimensional Morphology of Calcium Sulfide

**Figure 4:**
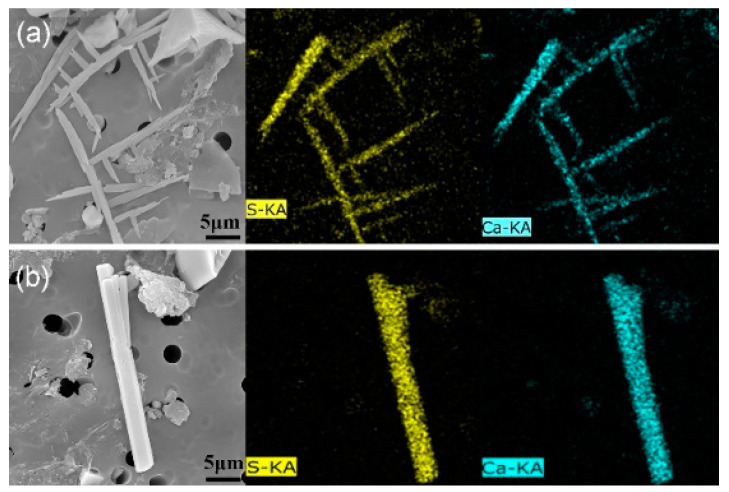
Element mapping of CaS inclusions in different morphologies. (**a**) Dendrite-like; (**b**) rod-like with branches.

Sulfide inclusions larger than 5 μm were analyzed and counted because they are more harmful to steel quality than small sulfides. Figure 4 shows the EDX scanning results, which shows that the observed inclusions are composed of Ca and S.

**Figure 5:**
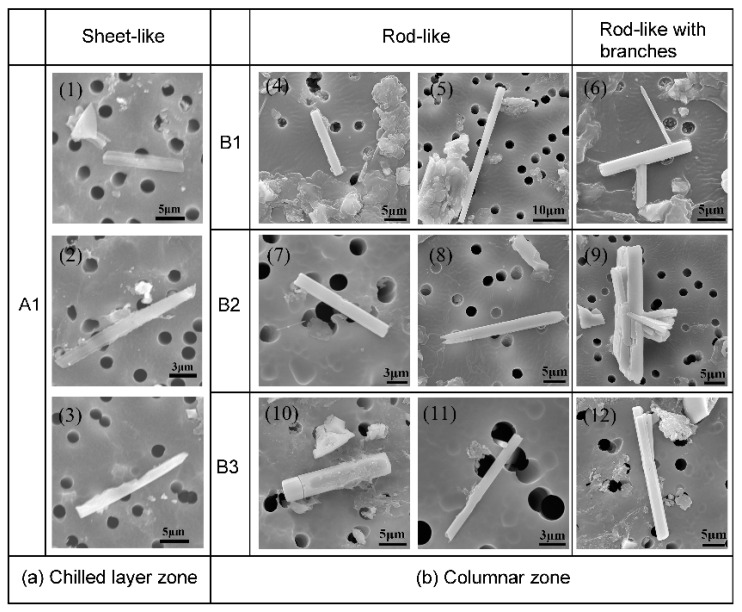
Typical morphologies of CaS inclusion in chilled layer zone (**a**) and columnar zone (**b**).

**Figure 6:**
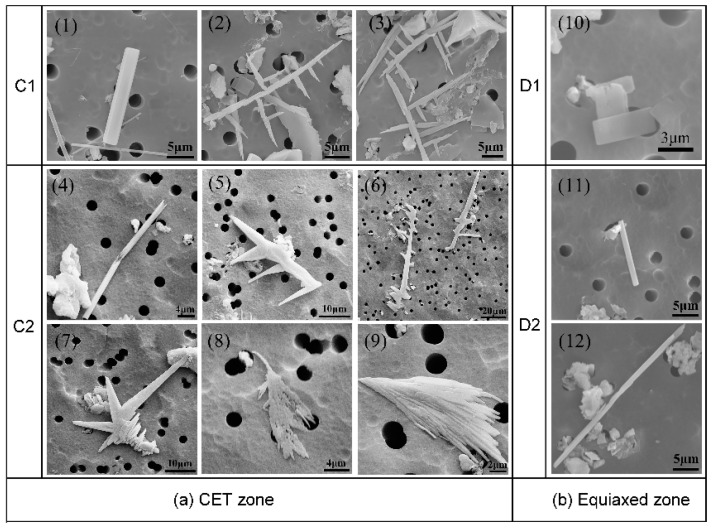
Typical morphologies of CaS inclusion in CET zone (**a**) and equiaxed zone (**b**). (1), (4), (11) and (12) are rod-like CaS; (2), (3), (5) and (6) are dendrite-like CaS; (7), (8) and (9) are irregular-shaped CaS; (10) is sheet-like CaS.

The three-dimensional morphologies of CaS inclusions in different regions of slab are shown in Figure 5 and Figure 6.

The morphologies of CaS inclusion are similar to the MnS inclusions, which has been reported early. Referring to the Sim and Oikawa classification [9,30], we categorized the 3D morphology of CaS inclusions into five groups: (1) rod-like CaS, (2) rod-like with branches CaS, (3) sheet-like CaS, (4) dendrite-like CaS, and (5) irregular-shaped CaS. The photomicrographs show that the rod-like with branches and the dendrite-like CaS has a complex structure, whose branches grow in a specific direction. The morphology of dendritic-like CaS is classified on the basis of dendrite sulfides in K. Oikawa’s classification [30].

**Figure 7:**
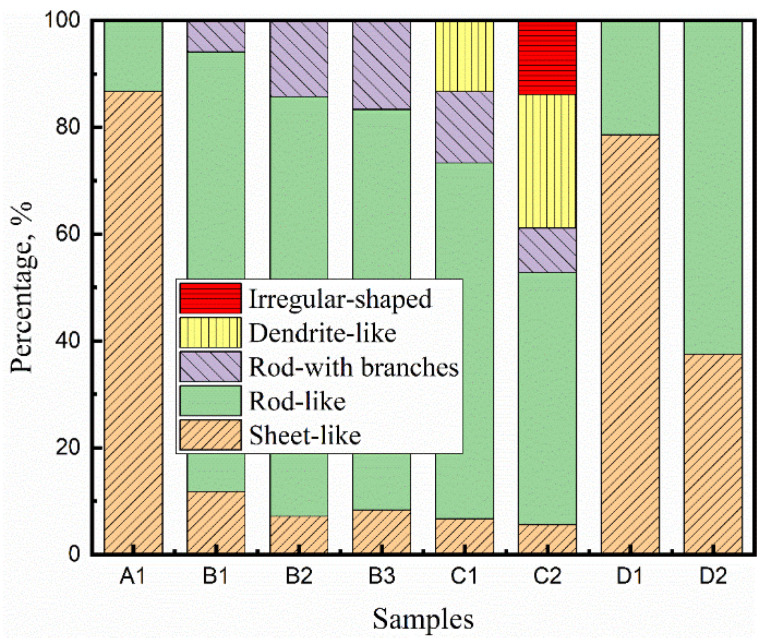
Number percentage of each inclusion morphology in each sample.

The morphologies of CaS inclusions vary with their locations in the slab. The percentages of each inclusion morphology in the samples are shown in Figure 7.

In the chilled layer zone, the sheet-like morphology of CaS inclusions is dominant. In the columnar zone, the rod-like and rod-like with branches CaS inclusions are observed. The percentage of rod-like branches CaS increased along the direction of columnar dendrites growth. The typical morphologies of CaS inclusions in the CET zone are shown in Figure 6a, where the dendrite-like and irregular-shaped CaS only existed in this zone. 

The morphology of CaS inclusions changes to sheet-like and rod-like in the equiaxed zone, as shown in Figure 6b. In the center of slab, the percentage of rod-like CaS is higher than the starting position of equiaxed zone, while the lower percentage is for sheet-like CaS. 

**Figure 8:**
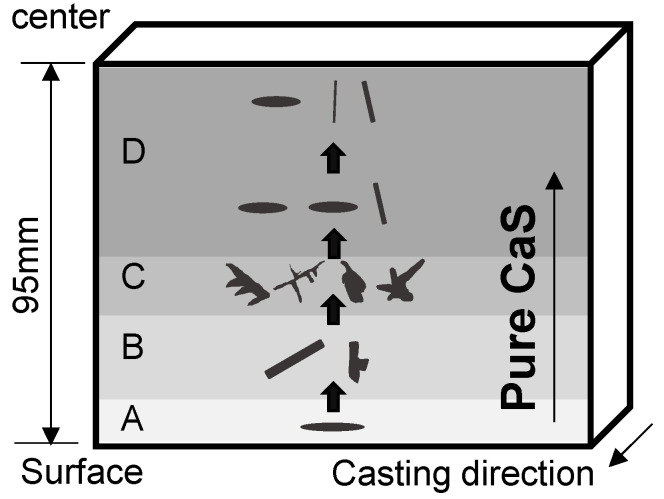
Schematic morphology distribution of CaS inclusions in slab (A is the chilled layer zone, B is columnar zone, C is CET zone, D is the equiaxed zone).

The morphology distribution of CaS inclusions in the slab is shown in Figure 8.

The morphology of CaS changed from sheet-like in the chilled layer zone to rod-like in the columnar zone, dendrite-like and irregular-shaped in the CET zone, and transformed to sheet-like and rod-like in the equiaxed zone.

### 3.3. Distribution of CaS Inclusions and Macrosegregation of Solutes

**Figure 9:**
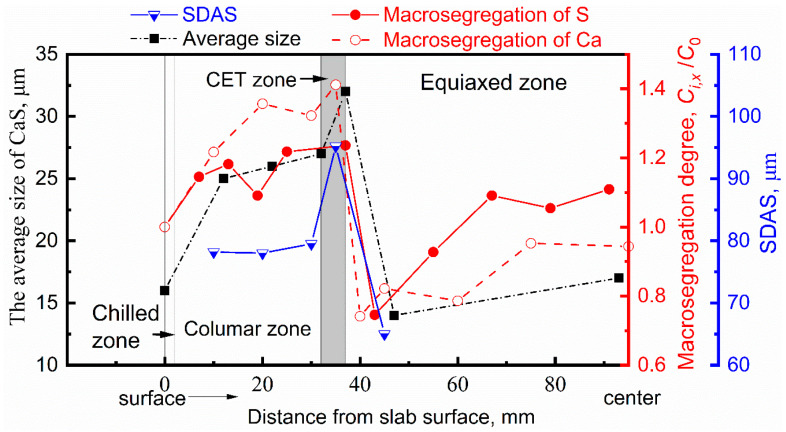
The size distribution of CaS inclusions and macrosegregation degree of S along the thickness of casting slab. The macrosegregation degree was defined by the ratio of concentration of solute at the x mm position from surface (*C_i,x_*) to initial liquid concentration (*C*_0_).

Figure 9 shows the distribution of macrosegregation degree of Ca and S, secondary dendrite arm spacing (SDAS), and the average size of CaS inclusions along the thickness direction of slab.

The background of the CET zone is marked as gray. These indices share a similar trend: increasing along the direction of dendrites growth, reaching the extrema in CET zone, then decreasing rapidly as soon as in the equiaxed zone.

The average size of CaS inclusions in CET zone is the largest, reaching 32 μm. The most severe positive macrosegregation degree of sulfur and calcium in the CET were 1.24 and 1.41, respectively. The average size of CaS inclusions quickly decreases to the minimum as soon as they leave the CET zone, where the most severe negative segregation of sulfur and calcium appears with the segregation degree of 0.75 and 0.74, respectively. The SDAS increased slowly in the columnar zone and reached the peak value of 95.2 μm in the CET zone.

The aspect ratio is defined as the ratio of the length to width of inclusions, which was considered as another representative value to characterize the morphology of sulfide inclusions. The aspect ratio of CaS inclusions in different regions of CC slab were summarized in Table 4, which represents another morphological characteristic for CaS inclusions.

The lower aspect ratio in the chilled layer zone, indicating that CaS inclusions in this region are finer and more rounded. The larger range of aspect ratio and standard deviation of CaS inclusions in the CET zone, indicating that the size and morphology of CaS inclusion are more diverse.

## 4. Discussion

### 4.1. Fundamentals for the Formation of Calcium Sulfide

During solidification process, the thermodynamic precipitation behavior of CaS inclusions is discussed as following. The reaction involved in CaS formation is, Equation (1):

(1) [Ca]+[S]=(CaS)

Equilibrium solubility product *K*_CaS_ (*a*_CaS_ = 1) [31] is given by Equation (2):

(2) $$\ln K_{CaS}=-\frac{65255}{T}+14.57$$

Actual concentration product *Q*_CaS_ is given by Equation (3):

(3) $$Q_{CaS}=f_{Ca}f_{S}C_{L,Ca}C_{L,S}$$

where, *a*_CaS_ is the activity of CaS, which was taken as unity in this calculation. *f_i_* is the activity coefficients of S and Ca, defined as Equations (4) and (5) *C_L,i_* is the concentration of solute in the residual liquid defined by Brody–Flemings equation [32].

(4) $$\mathrm{lg}f_{i}=\left(2538/T_{S-L}-0.355\right)\mathrm{lg}f_{i\left(1873K\right)}$$

where, *f_i_*_(1873K)_ is activity coefficients of S and Ca which were calculated by Factsage, 0.54 and 9.86 × 10^−6^, respectively. *T_S-L_* is temperature at the solid–liquid interface given by:

(5) $$T_{S-L}=T_{0}-\frac{T_{0}-T_{L}}{1-f_{S}\frac{T_{L}-T_{S}}{T_{0}-T_{S}}}$$

where, *T*_0_ is the melting point of pure iron, *T_L_* is liquidus temperature of steel, *T_S_* is solid temperature of steel, respectively.

**Figure 10:**
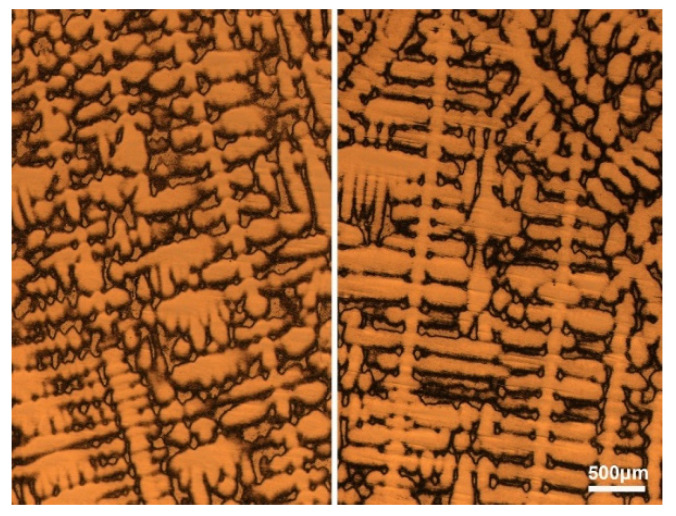
Typical dendrite morphologies in the columnar zone.

Following the Brody–Flemings model, the changes in the mass fraction of Ca and S in the liquid phase with the solidified fraction are defined in Equation (6):

(6) $$C_{L}=C_{0}{\left(1-\left(1-2\alpha k\right)f_{S}^{\prime }\right)}^{\left(k-1\right)\left(1-2\alpha k\right)},\alpha =\frac{4D_{S}t_{f}}{\lambda^{2}}$$

where *C*_0_ is the initial liquid concentration, α is the back-diffusion parameter, *k* is the equilibrium partition coefficient, $f_{S}^{\prime }$ is the solid fraction, *D_S_* is the diffusion coefficient of solute in the solid phase in cm^2^s^−1^, *t_f_* is the local solidification time in seconds, and *λ* is the secondary dendtrite arm spacing in cm, estimated through measurements of average secondary dendrite arm spacing from Figure 10.

Local solidification time in Equation (6) is defined as Equation (7):

(7) $$t_{f}=\frac{T_{L}-T_{S}}{C_{R}}$$

where *T_L_* and *T_S_* are liquidus and solidus temperatures of steel, respectively. 

The cooling rate (*C_R_*) is defined as in Equation (8):

(8) $$\lambda =688\times {\left(60C_{R}\right)}^{-0.36}$$

The condition for precipitation of pure CaS inlcusions is given by Equation (9):

(9) $$Q_{CaS}\geq K_{CaS}$$

**Figure 11:**
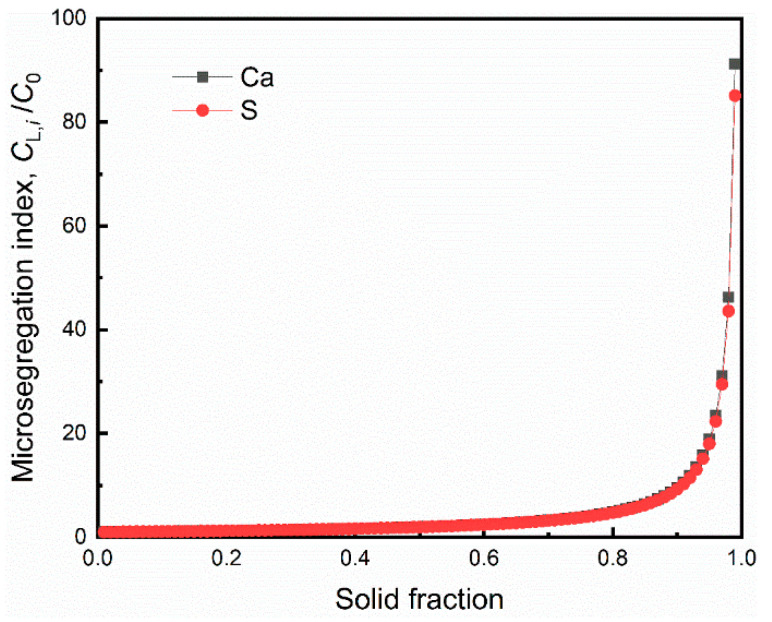
The interdendrite solute segregation during solidification. The ratio of interdendritic solute concentration to initial solute concentration was defined as the microsegregation degree (*C_L,i_*/*C*_0_).

The parameters and conditions for the calculation are listed in Table 5. Figure 11 presents the interdendrite solute segregation, with the solid fraction during solidification.

**Figure 12:**
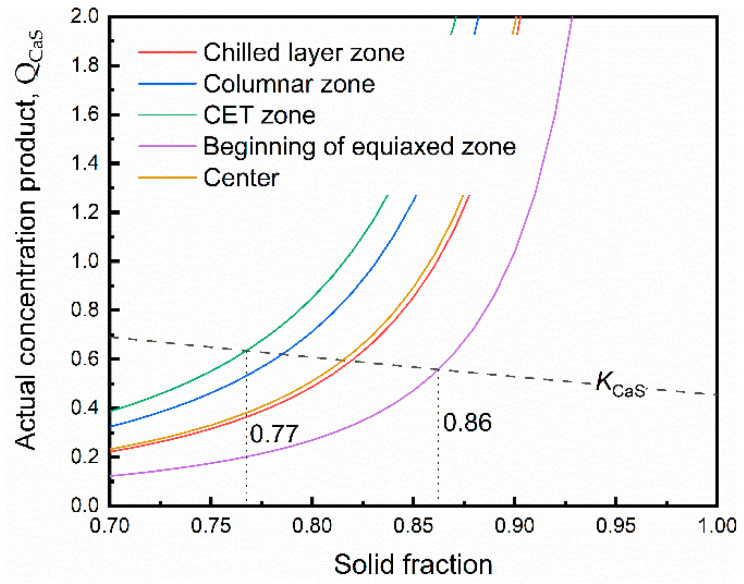
Formation of CaS in different zone.

At the final stage of solidification process, the solute of S and Ca showed a strong tendency of segregation because of the small equilibrium partition coefficient (*k*). Figure 12 presents the variation of actual concentration product for the interdendritic liquid and equilibrium solubility product with solid fraction.

At the initial stage of solidification, the CaS particles are difficult to precipitate in the liquid phase because of the low concentration product of Ca and S. The precipitation of CaS occurs at the front of solidification with the consumption of excess solutes when the actual concentration product (*Q*_CaS_) reaches the equilibrium solubility product (*K*_CaS_). The results indicated that CaS inclusions were precipitated at the end of solidification due to the microsegregation of S and Ca, and the precipitated CaS particles are enriched in the residual liquid phase. As the macrosegregation degree of S increases, the CaS particle precipitates earlier (solid fraction is 0.77 in CET zone). The CaS particles earlier precipitated in CET zone have more time to collision and growth, resulting in the larger size of CaS inclusions. It is one of the reasons that the largest size of CaS inclusion precipitated in the CET zone.

**Figure 13:**
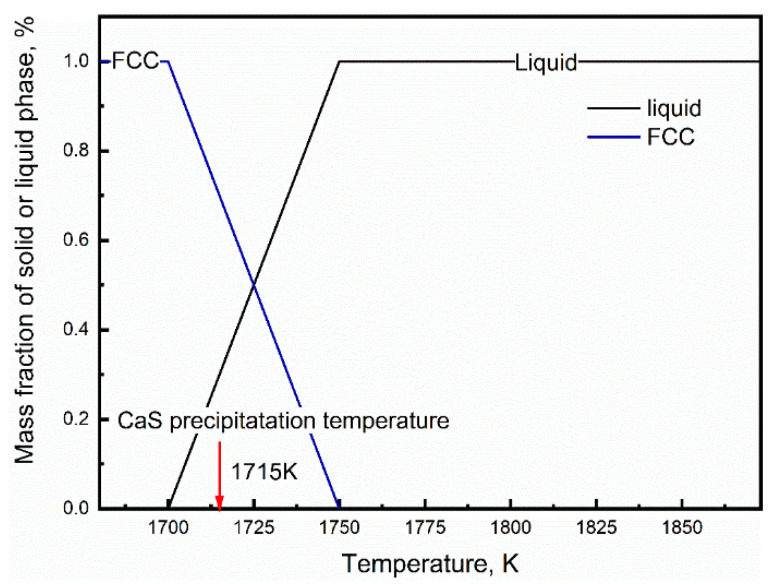
Change of phase fraction with solidification.

The solidification process of molten steel is a process of nucleation and growth. At the beginning of crystallization, tiny grains form in the liquid metal and keep growing up until the liquid phase disappears. Figure 13 shows the change of phase fraction during solidification, only austenite (*γ*-Fe) phase formed during the entire solidification process.

**Figure 14:**
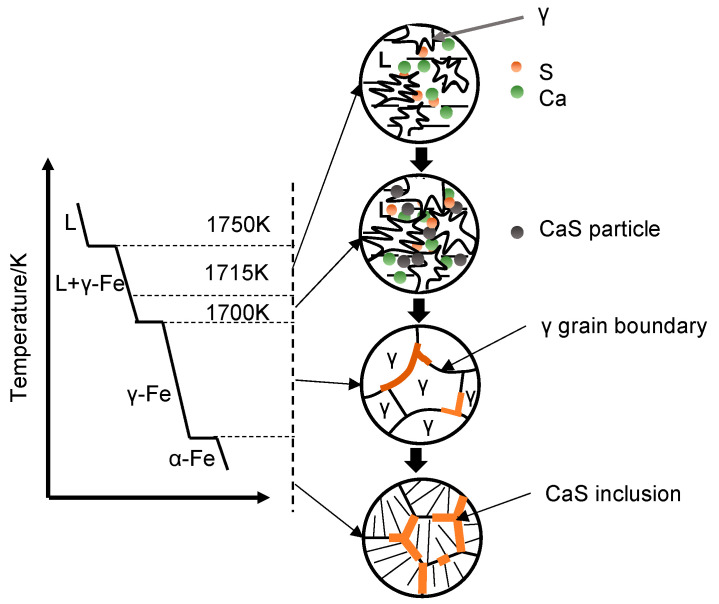
Schematic illustration of formation of CaS during steel solidification.

**Figure 15:**
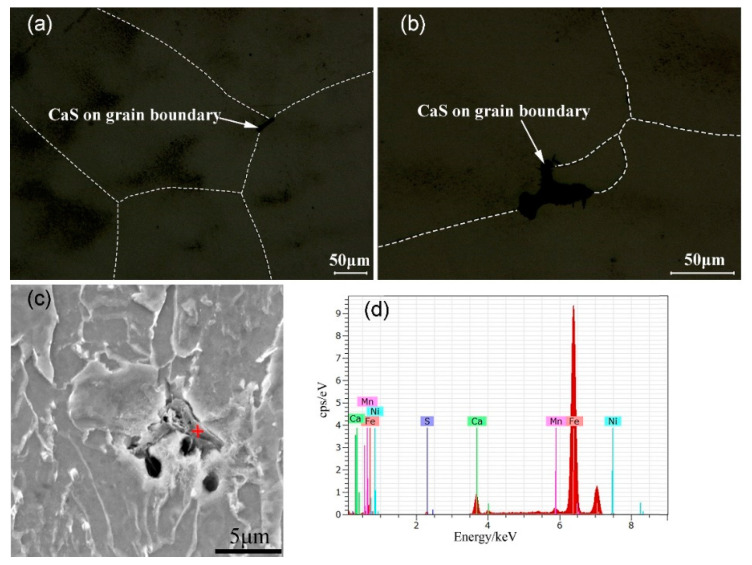
CaS inclusions in grain boundaries found in the CET zone, where the red cross is the location of EDX analysis. (**a**) CaS in the boundary of austenite grain, (**b**) CaS in the boundary of austenite grain, (**c**) illustration of EDX location, (**d**) results of energy spectrum analysis.

Along with the temperature decreasing, the austenite crystal grows with solute rejection, resulting in solute enrichment in the residual liquid phase. The residual liquid phase finally gathered at the *γ* grains boundary with the growth of *γ* crystals. As the temperature decreases, CaS particles nucleated in the residual liquid and finally precipitated along the austenite grain boundaries. Figure 14 illustrates the formation of CaS during solidification. Figure 15 proved the correctness of CaS inclusions existing in the boundary of austenite grains. 

### 4.2. Influence of Macrostructure on the CaS Inclusions

According to the experimental results, the characteristics of the CaS inclusion in the transverse section of slab were related to the solute concentration and solidification structure of local positions. During the solidification of liquid metal, the residual liquid phase, which is solute-rich due to solute rejection, flows through the channels between the dendrites under the effect of physical changes in the melt. The liquid flow causes the macrosegregation and affects the growth of inclusion in the cross section of the slab.

**Figure 16:**
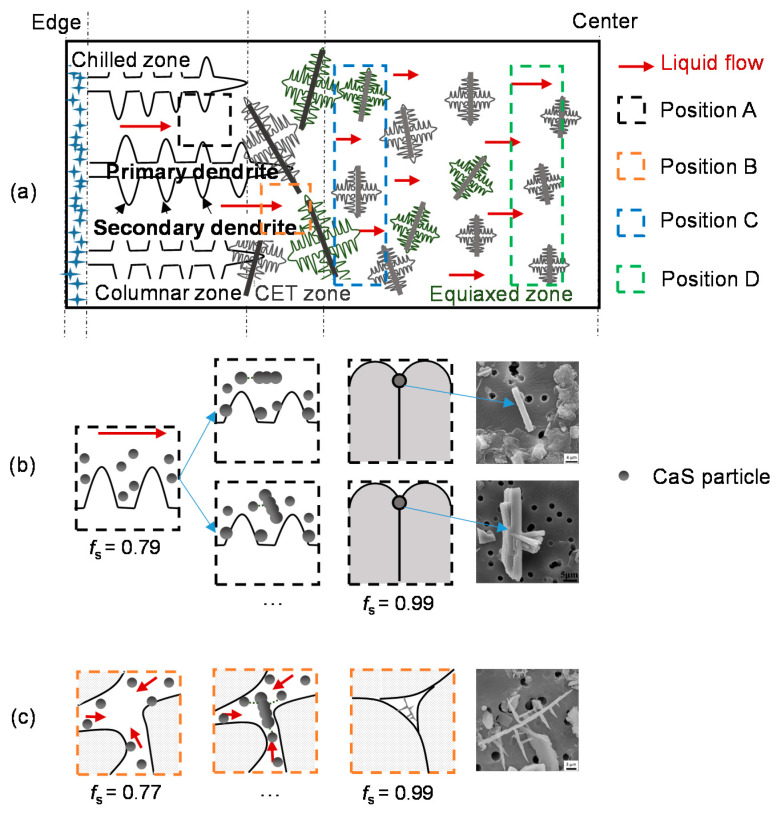
The growth of CaS inclusion in different macrostructure. (**a**) The view of macrostructure of slab; (**b**) the schematic diagram of CaS growth in columnar zone (position A); (**c**) the schematic diagram of CaS growth in CET zone (position B).

Because of the higher cooling rate in the mold, a thin chilled layer with fine equiaxed grains forms near the surfaces rapidly, shown in Figure 16a. CaS inclusions are rapidly generated in smaller size, and there is almost no liquid flow in the chilled layer zone.

Therefore, the morphology of CaS inclusions was determined by the austenite grain boundaries, resulting in formation of sheet-like CaS. The sulfur and calcium content were regarded as initial content in this position. 

**Figure 17:**
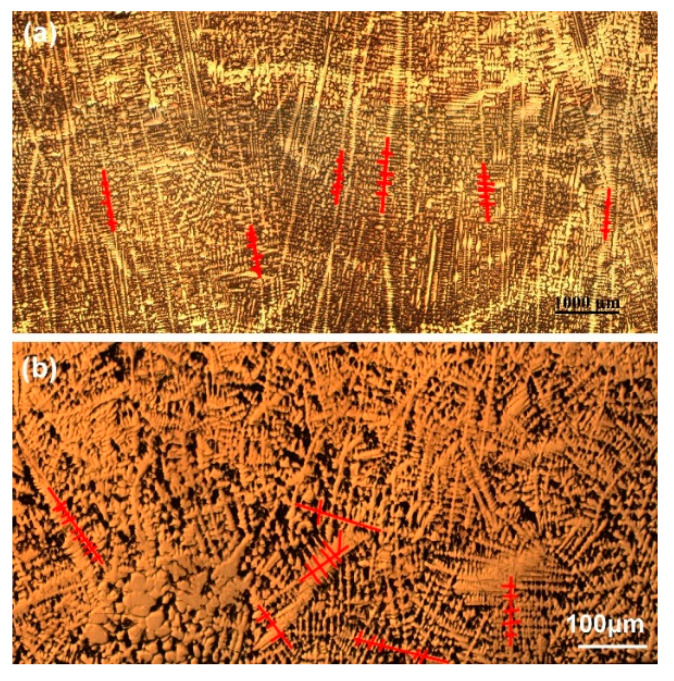
Morphology of macrostructure of slab. (**a**) Columnar zone; (**b**) CET zone.

As the grain size gradually increases, columnar crystals begin to appear. The dendrites grow uniformly toward the slab center in the columnar zone, as shown in Figure 17a, leading to the consistency liquid flow direction and small flowing resistance of liquid phase.

The flow of solute-enriched liquid phase causes the positive macrosegregation in the columnar zone, which increased with the columnar dendrites grows. The crystal grows on the side facing the liquid flow, so that the nucleated CaS particles tend to collide and grow parallel to the flow direction in the columnar zone. As a consequence, the growth of CaS inclusions were easily constrained by columnar dendrite growth and liquid flow, causing a rod-like and occasionally a rod-like with branches morphology. The schematic illustration of CaS growth in columnar zone is shown in Figure 16b.

CET formed when the columnar and equiaxed grains coexist in the macrostructure [28,29]. The results of Section 3 show that CET is the key transition position of CaS inclusions, where both the SDAS and solutes segregation reach the maximum. The dendrites are randomly arranged and coarse in CET zone, as shown in Figure 17b. The solute-rich liquid flow from columnar zone is blocked by the staggered dendrites in the CET zone [36], so that more solutes can be accumulated in the clearance of the coarse dendrites and the direction of liquid flow can be changed in this region. The accumulation of solutes causes an extreme positive macrosegregation in the CET zone. Furthermore, CaS inclusions with special shapes, for instance dendrite-like and irregular-shaped ones, only exist in the CET zone. The complex dendrite morphology lead to the irregularity of liquid flow, causing the random collision of CaS particles and promoting the irregular growth of CaS inclusions. The coarse dendrite structure provides a larger space for the growth of CaS particles, resulting in a large CaS inclusion. Thus, in columnar zone and CET zone, the morphology of CaS inclusions was mainly decided by the direction of liquid flow. The schematic illustration of CaS growth in columnar zone is shown in Figure 16c.

The liquid phase can pass through the equiaxed zone with smaller resistance and better permeability because the equiaxed grains were smaller size, larger amount, and less interlaced than in the CET zone, resulting in the lower rate and more branches of liquid flow, as shown in Figure 16a. The solute-rich liquid phase flowed towards the slab center, contributing to form the negative segregation at the beginning of equiaxed zone (Position C) and positive segregation in the center of slab (Position D). The rate of liquid flow in the Position C was lower than Position D, the grain boundaries played a main factor on the morphology, resulting in the higher percentage of sheet-like CaS inclusions in position C, while the higher percentage of rod-like CaS inclusions is in position D.

## 5. Conclusions

The following conclusions were obtained by investigation of the CaS morphology in the cross-section of Ni20Mn6 steel slab:

(1) Five types of CaS morphologies were found and defined as (1) rod-like CaS, (2) rod-like with branches CaS, (3) sheet-like CaS, (4) dendrite-like CaS, and (5) irregular-shaped CaS.
(2) The morphology of CaS changed from sheet-like near surface to rod-like in the columnar zone, dendrite-like, and irregular-shaped in the columnar-to-equiaxed transition (CET) zone and changed back to rod-like and sheet-like in the equiaxed zone.
(3) The average size of CaS inclusions increases along the direction of dendrites growth, reaching the extrema value of 32 μm in CET zone, then decreases rapidly as soon as entering the equiaxed zone. The same trend was found for macrosegregation degree of sulfur and calcium, whose maximum values were also found in the CET zone, reaching 1.24 and 1.41, respectively. The liquid flow was blocked by the interlaced and coarse dendrites in the CET zone, causing the segregation of solutes.
(4) Thermodynamic calculation showed that CaS inclusions were formed in the clearance of dendrite at the end of solidification process due to the microsegregation of sulfur and calcium. Only the precipitation of austenite phase runs through the entire solidification process, prior to the precipitation of CaS particles. Thus, the CaS inclusions precipitated along the austenite grain boundaries.
(5) The sheet-like CaS inclusion in the chilled layer zone was determined by the austenite grain boundaries due to the higher cooling rate in the mold. The aligned dendrites in the columnar zone caused the consistent direction of liquid flow, resulting in the rod-like and, occasionally, rod-like CaS inclusions. The joint effect of complex structure and larger space promoted the precipitation of CaS inclusions with large size and irregular shape in the CET zone. The coexistence of sheet-like and rod-like CaS inclusions was decided by the austenite grain boundaries and the smaller and dense liquid flow.

## Figures and Tables

**Table 1:** Chemical compositions of casting slab, wt.%.

C	Mn	P	S	Si	Ni	Cr	Cu	Mo	Al	Ca	T.O
0.019	5.96	0.012	0.0005	0.18	19.86	0.25	0.04	0.04	0.008	0.0008	0.0012

**Table 2:** Sampling location of each zone in casting slab.

Sample	A1	B1	B2	B3	C1	C2	D1	D2
Distance from surface/mm	0	2	12	22	32	0~45	47	93

**Table 3:** Width of each zone of slab macrostructure (mm).

No.	Macrostructure	Width/mm	Area Ratio/%
a	Chilled layer zone	2	2.1
b	Columnar zone	30	31.6
c	CET zone	5	5.3
d	Equiaxed zone	58	61

**Table 4:** Aspect ratio of CaS inclusions in different regions of continuous casting (CC) slab.

Aspect Ratio Statistics	Chilled Layer Zone	Columnar Zone	CET Zone	Equiaxed Zone
Aspect ratio	5.9–12.2	5.2–22.9	6.3–58.2	7.3–44.8
Mean aspect ratio	7.8	10.6	19.2	15.56
Standard deviation	5.86	1.88	12.93	10.94

**Table 5:** Conditions for the calculation of CaS formation [33,34,35].

Element	*k*	*D_S_* × 10^4^, m^2^/s
S	0.035	2.4 exp(−223,426/R*T*)
Ca	0.02	0.055 exp(−249,366/R*T*)
Cooling rate, *C_R_*		7 °C/s
Secondary dendrite arm spacing, *λ*		77 μm

where R is the gas constant, *T* is the temperature.
